# HIV Neuroinflammation: The Role of Exosomes in Cell Signaling, Prognostic and Diagnostic Biomarkers and Drug Delivery

**DOI:** 10.3389/fcell.2021.637192

**Published:** 2021-04-01

**Authors:** Supriya D. Mahajan, Nigel Smith Ordain, Hilliard Kutscher, Shanta Karki, Jessica L. Reynolds

**Affiliations:** ^1^Department of Medicine, Jacobs School of Medicine and Biomedical Sciences, University at Buffalo, Buffalo, NY, United States; ^2^Institute for Laser, Photonics and Biophotonics, University at Buffalo, The State University of New York, Buffalo, NY, United States; ^3^Department of Anesthesiology, State University of New York at Buffalo, Buffalo, NY, United States

**Keywords:** HIV neuroinflammation, exosomes, signaling, delivery, blood-brain barrier

## Abstract

Fifty to sixty percent of HIV-1 positive patients experience HIV-1 associated neurocognitive disorders (HAND) likely due to persistent inflammation and blood–brain barrier (BBB) dysfunction. The role that microglia and astrocytes play in HAND pathogenesis has been well delineated; however, the role of exosomes in HIV neuroinflammation and neuropathogenesis is unclear. Exosomes are 50–150 nm phospholipid bilayer membrane vesicles that are responsible for cell-to-cell communication, cellular signal transduction, and cellular transport. Due to their diverse intracellular content, exosomes, are well poised to provide insight into HIV neuroinflammation as well as provide for diagnostic and predictive information that will greatly enhance the development of new therapeutic interventions for neuroinflammation. Exosomes are also uniquely positioned to be vehicles to delivery therapeutics across the BBB to modulate HIV neuroinflammation. This mini-review will briefly discuss what is known about exosome signaling in the context of HIV in the central nervous system (CNS), their potential for biomarkers as well as their potential for vehicles to deliver various therapeutics to treat HIV neuroinflammation.

## Introduction

The lifespan of patients with HIV-1 has significantly improved due to combination therapy with highly active antiretroviral therapy (HAART). However, HIV-1 associated neurocognitive disorders (HANDs) develop in 50–60% of patients and cause significant morbidity in this population (Hogan and Wilkins, 2011; Valcour et al., 2011). HAND is classified into three categories, including “asymptomatic neurocognitive impairment, mild neurocognitive disorder, or HIV-associated dementia” (Anthony and Bell, 2008; Ferrell and Giunta, 2014; Malik and Eugenin, 2016; Chivero et al., 2017; Guo and Buch, 2019; Mitra and Sharman, 2020). Confusion, headaches, cognitive motor impairment, and anxiety disorders are common and increase health-care costs (Hogan and Wilkins, 2011; Valcour et al., 2011). Central nervous system (CNS) dysfunction induced by multiple factors likely contributes to the progression and severity of HAND (Anthony and Bell, 2008; Ferrell and Giunta, 2014; Malik and Eugenin, 2016; Chivero et al., 2017; Guo and Buch, 2019). Microglia, CNS-resident mononuclear phagocytic cells permissible to HIV infection, are implicated in the neuropathogenesis of HIV (Schnell et al., 2001; El-Hage et al., 2006, 2015; Cherry et al., 2014; Dever et al., 2015; Joseph et al., 2015; Tang and Weidong, 2016; Sillman et al., 2018). Additionally, the largest population of cells in the CNS, astrocytes, are sources of persistent inflammation during HIV infection (Brack-Werner, 1999; Hong and Banks, 2015; Li et al., 2016; Rodriguez et al., 2017; Lutgen et al., 2020). While much is known about microglia cells and astrocytes in HIV neuroinflammation, the role that exosomes play in HIV neuropathogenesis has not been completely elucidated and is a rapidly developing field that may further contribute to the understanding of HIV neuroinflammation. This mini-review will succinctly discuss the role exosomes in HIV induced toxicity in the CNS and the potential of exosomes to deliver therapeutics to the CNS for HIV neuroinflammation.

## Extracellular Vesicles

Extracellular vesicles (EVs) are characterized as exosomes, macrovesicles, and apoptotic bodies (Ohno et al., 2013; Edgar, 2016; Fujita et al., 2018; Kalluri and LeBleu, 2020). Exosomes, in particular, are 50–150 nm phospholipid bilayer membrane vesicles. Exosomes are synthesized by invagination of the plasma membrane forming intracellular multivesicular bodies (MVBs), which contain intraluminal vesicles (ILVs). ILVs are then secreted as exosomes through the fusion of the MVB with the plasma membrane and exocytosis (For in-depth review on exosome biogenesis, see Edgar, 2016; Ha et al., 2016; Kalluri and LeBleu, 2020). Once released into the extracellular milieu, exosomes interact with other cells resulting in physiological changes (Ohno et al., 2013; Kalluri and LeBleu, 2020). Exosomes under normal and pathological conditions have multiple biological functions including autocrine, paracrine and endocrine communication, and immunoregulatory potential (Eldh et al., 2010). Multiple cells including but not limited to B and T lymphocytes, macrophages, astrocytes, microglia, myocytes, adipocytes, and Schwann cells produce exosomes, and exosomes are also detected in urine, serum, and saliva (Lenassi et al., 2010; Nanjundappa et al., 2011; Tang et al., 2018; Pulliam et al., 2019). Exosomes express surface markers such as LAMP1, LAMP2b, and ALIX-1 proteins, and CD9, CD63, and CD81 (Edgar, 2016; Liu et al., 2019). In addition, exosomes are comprised of a variety of proteins such as heat shock proteins (HSP70, HSP90) as well as genetic materials such as microRNA (miRNA) and messenger RNA (mRNA). The content of exosomes [proteins, mRNAs, microRNAs (miRNAs), and signaling molecules] have potential impact as effectors on distant cells and tissues (Ludwig and Giebel, 2012; Ohno et al., 2013; Kalluri and LeBleu, 2020) and consequently provide a greater opportunity to identify potential biomarker molecules. Exosomes were often overlooked by researchers until findings showed that they have the potential for immunomodulatory effects (Ludwig and Giebel, 2012; Fujita et al., 2018).

## HIV and Exosomes

### Peripheral Studies

Extensive work has been done investigating exosomes in the context of HIV in plasma and peripheral immune cells (Patters and Kumar, 2018). Overall studies have shown that exosomes facilitate the transport of viral proteins (i.e., Tat protein and Nef) and host proteins (i.e., pro- and anti-inflammatory cytokines/chemokines, markers of oxidative stress) and facilitate viral dissemination (Patters and Kumar, 2018). A few key studies are highlighted below. Studies have shown that HIV structural and accessory proteins Gag (Booth et al., 2006; Fang et al., 2007; Columba Cabezas and Federico, 2013) and Nef (Lenassi et al., 2010; Shelton et al., 2012; Aqil et al., 2013, 2014; Arenaccio et al., 2014a) are secreted in exosomes from various cells types. The HIV-1 accessory protein Nef, integral to HIV-associated immunopathogenesis, was found within plasma-derived exosomes of HIV-1-infected patients with HAND (Khan et al., 2016). Studies have shown that unspliced HIV-1 RNA sequences encoding for Gag can be incorporated in exosomes (Columba Cabezas and Federico, 2013). The exosome pathway in macrophages plays a major role in HIV budding (Nguyen et al., 2003) and facilitates viral infection of other cells (Kadiu et al., 2012). Dendritic cells transmit HIV-particles to T cells *via* exosomes (Wiley and Gummuluru, 2006; Izquierdo-Useros et al., 2010, 2012; Nanjundappa et al., 2011; Wang C. et al., 2013; Naslund et al., 2014). HIV-1 replication occurs in CD4 T lymphocytes exposed to exosomes derived from HIV-1-infected cells (Arenaccio et al., 2014a,b). However, other studies have shown that HIV-1 is produced independently of exosomes in CD4 + T lymphocytes (Park and He, 2010). Additionally, studies have shown that exosomes from HIV-infected patient sera contain HIV trans-activation response (TAR) element; the number of copies/ml were reduced in exosomes of HAART patients or long-term non-progressors (Narayanan et al., 2013). In macrophages, TAR RNA-exosomes also significantly increases the levels of interleukin-6 (IL-6) and tumor necrosis factor-β (TNF-β) (Chen et al., 2018). These studies demonstrate the role of exosomes in modulating HIV signaling in the periphery.

### CNS Exosomes and HIV

Preliminary studies have been done investigating the interactions of HIV proteins and exosomes and their subsequent effects on cells of the CNS; however, much work is still to be done to understand the role of exosomes in HIV neuroinflammation. Other studies have also examined the potential of exosomes as biomarkers for HAND. Below, a select few key studies are emphasized.

### Biomarkers

Due to their diverse intracellular content, exosomes are poised to provide diagnostic and predictive information that will greatly enhance the development of new therapeutic interventions. Characterization of the cerebral spinal fluid (CSF) exosome proteome found that certain proteins (concentration and number) detected in exosome fractions were higher in HIV + subjects with HAND compared to those without HAND (see Table 1A). This study suggests that CSF exosomes may be a valuable source of biomarkers (Guha et al., 2019a), which is further supported by findings from this same laboratory that a novel CSF exosome biomarker, neurofilament light chain, correlates with neurocognitive impairment in cART-treated HIV-positive individuals (Guha et al., 2019b). A study by Sun et al. (2019) found that proteins from plasma neuron-derived enriched exosomes (NDEs) differ in HIV infection alone and cognitive impairment between men and women suggesting mechanistic sex differences (Sun et al., 2019). Potential NDE proteins that may represent biomarkers are shown in Table 1B. Combined, these studies support the potential role of exosomes for diagnostic biomarkers of HAND. Further studies are necessary to determine the possibility of exosomes as predictive biomarkers of disease progression.

**TABLE 1:** **(A)** Potential Biomarkers of HAND; **(B)** potential biomarkers in males and females with mild impairment.

A. Potential biomarkers of HAND		

Category/function	Proteins	Citations
Example of some proteins in exosome fractions that were higher in HIV + subjects with HAND compared to those without HAND		
Inflammatory/immune response	Annexins, C-reactive protein, enolase, human leukocyte antigen (HLA),	Guha et al., 2019a
Stress response proteins	Parkinsonism associated deglycase Peroxiredoxins, alpha-synuclein, synuclein, vimentins	Guha et al., 2019a
Neuronal	Cell adhesion, ankyrin-binding protein, Neuroplastin, Neurexins	Guha et al., 2019a
Astrocyte proteins	Aldehyde dehydrogenase, glial fibrillary acidic *protein*, glutamine synthetase, calcium-binding peptide	Guha et al., 2019a
Choroid plexus protein	ATPase Na + /K + transporting subunit alpha 2, ATP synthase, H + transporting, mitochondrial F1 complex, beta polypeptide	Guha et al., 2019a

**B. Potential biomarkers in males and females with mild impairment**		

**Category/function**	**Proteins**	**Citations**

Example of proteins from NDE from women with mild impairment that were significantly decreased		
Lysosomal cysteine protease	Cathepsin S	Sun et al., 2019
Microtubule-associated protein	Total tau	Sun et al., 2019
Cell recognition and cell–cell adhesion	Neuronal cell adhesion molecule	Sun et al., 2019
Cell adhesion molecule	Contactin-5	Sun et al., 2019
Example of proteins from NDE that were increased from cognitively impaired men		
Blood coagulation/fibrinolysis, inflammation	Carboxypeptidase M	Sun et al., 2019
Calcium-dependent cell adhesion proteins	Cadherin 3	Sun et al., 2019
Endoplasmic reticulum (ER) stress-inducible neurotrophic factor.	Mesencephalic astrocyte-derived neurotropic factor	Sun et al., 2019

### Exosomes and Nef

Nef, a HIV-1 regulatory protein, stimulates viral infectivity by facilitating early events in the HIV-1 life cycle (Geyer et al., 2001; Buffalo et al., 2019). Transfecting astrocytes or microglia with Nef results in the presence of Nef in exosomes of respective cells (Raymond et al., 2016; Puzar Dominkus et al., 2017). Nef expression in human astrocytes increases the secretion of Nef-containing exosomes up to 5.5-fold (Puzar Dominkus et al., 2017). Nef-containing exosomes are taken up by neurons, inducing multiple forms of stress, including oxidative, which suppresses neuron action potentials (Sami Saribas et al., 2017). Furthermore, Nef-transfected microglia-release Nef + exosomes that disrupt the integrity and permeability of the blood–brain barrier (BBB) (Raymond et al., 2016). The expression of the BBB tight junction protein, ZO-1, decreases following exposure to microglia-derived exosomes containing Nef. Microglia exposed to exosomes containing Nef had increases in the levels of IL-12, IL-8, IL-6, RANTES, and IL-17A (Raymond et al., 2016). Interestingly, in neuroblastoma cells, Nef protein within plasma exosomes induces production and secretion of amyloid beta protein. This study demonstrates the role that peripheral exosomes may play in neuroinflammation or HAND, as amyloid beta (Aβ) production may increase the severity of HAND (Khan et al., 2016). Elegant studies from Michal Toborek’s laboratory demonstrate that HIV infection accelerates the release of brain endothelial exosomes and alters exosome-Aβ levels. These exosomes transfer Aβ to astrocytes (András et al., 2017). They have also found that HIV along with Aβ alter brain endothelial exosomes proteome, which disrupts networks and functional interactions (András et al., 2017, 2020). Overall, this body of work demonstrates the potential contribution of Nef-containing exosomes in the development of neuroinflammation associated with HIV.

### Exosomes and Tat

HIV Trans-activating regulatory protein (Tat) stimulates transcription from the viral long terminal repeat promoter (Das et al., 2011). Buch’s laboratory has done pioneering work in Tat-induced expression of microRNAs (miRNA) in exosomes. miRNAs are non-coding RNAs of 19–22 nucleotides that regulate gene expression and cell homeostasis (Hamrick et al., 2010; Smith-Vikos and Slack, 2012; Kim et al., 2014). HIV Tat increases the release of exosomes containing miR-29b (Hu et al., 2012) in astrocytes. Neuronal cells treated with these exosomes had decreases in neurotrophic factor platelet-derived growth factor expression, as well as a decrease in viability of neurons (Hu et al., 2012). Furthermore, Tat-stimulated astrocytes release miR-9 in exosomes that are engulfed by microglia. This prompts a change in microglia migratory phenotype (Yang et al., 2018). In astrocytes and neurons, HIV-1 Tat protein significantly upregulates the number of exosomes containing miR-132. Tat-expressing astrocytes release exosomes with miR-132 that are taken up by neurons, inducing neurite shortening and increases in neurotoxicity (Rahimian and He, 2016). Engineered exosomal Tat adeno-associated viruses significantly reduce the expression levels of synaptophysin (synaptic marker) in the brain of mice, demonstrating HIV-1 Tat protein attributes to synaptic damage (Tang et al., 2020). Exosomes from CSF samples were positive for Tat protein and retained transactivation activity, indicating that Tat present in CSF is functional and likely found in exosomes, further implicating the role of exosomal Tat in neurotoxicity (Henderson et al., 2019). Cumulatively, these data provide new mechanistic insights in Tat proteins signaling and neurotoxicity and demonstrate the potential role of exosomes in HIV signaling both in HIV-related neuroinflammation and neurotoxicity. Additional studies are necessary to further demarcate the role of exosomes in Tat function, transport, and association with HAND.

### Delivery of Therapeutics to the CNS Using Exosomes

**FIGURE 1:**
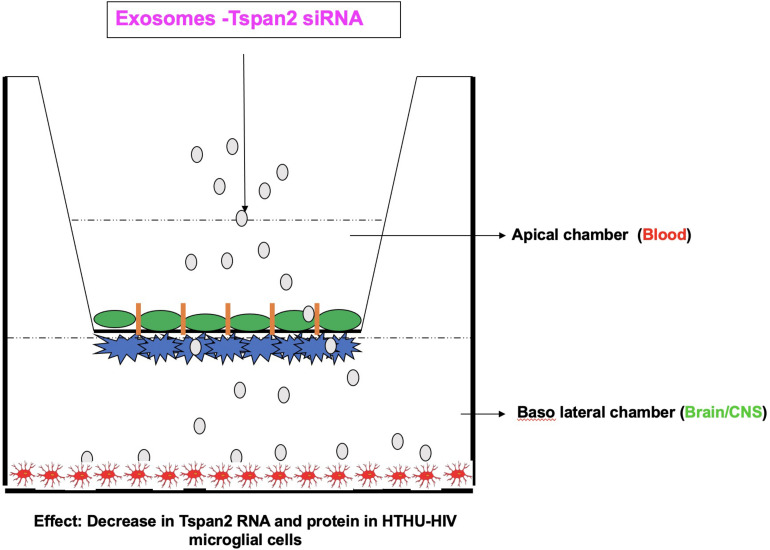
Blood–brain barrier model demonstrating proof of concept of exosome delivery of Tspan2 across the BBB to latently infected microglia on the brain side. Schematic adapted from Reynolds and Mahajan (2020).

Targeting and delivering therapeutics to the CNS has been a longstanding challenge in biomedical research. The BBB separates circulating blood from the brain extracellular fluid. It is a highly selective permeable barrier that regulates diffusion of molecules into the brain (Serlin et al., 2015; Reynolds and Mahato, 2017; Reynolds and Mahajan, 2020). Only small, lipophilic compounds (<400–500 Da) or hydrophobic molecules (e.g., O_2_, CO_2_, hormones) can cross the BBB (Reynolds and Mahato, 2017; Reynolds and Mahajan, 2020). The BBB contains endogenous, receptor-mediated, and efflux transporters. Endogenous transporters mediate selective uptake of water-soluble nutrients (e.g., glucose and amino acids), whereas, receptor-mediated transporters mediate the transport of large molecules [e.g., insulin and transferrin (Tf)]. Furthermore, efflux transporters [e.g., P-glycoprotein (Pgp)] pump substrates out of the brain (Abbott, 2004, 2005; Reynolds and Mahato, 2017; Reynolds and Mahajan, 2020). Multiple research efforts have been devoted to develop ways to increase the passage of therapeutics across the BBB and to increase the bioavailability of drugs in the CNS. Exosomes have characteristics of effective drug-delivery vehicles (van Dommelen et al., 2012; Lai et al., 2013; Aryani and Denecke, 2016; Ha et al., 2016). The structure of exosomes is a lipid bilayer membrane that allows for drug loading. The exosome lipid membrane surrounds a hydrophilic core (Frydrychowicz et al., 2015; Luan et al., 2017). Therapeutic agents are incorporated into exosomes through active or passive encapsulation (Luan et al., 2017). Diverse therapeutic agents have been loaded in exosomes such as small interfering RNA (siRNA) or small molecules (Alvarez-Erviti et al., 2011; El Andaloussi et al., 2013; Wang C. et al., 2019; Wang H. et al., 2019; Reynolds and Mahajan, 2020). Exosomes are slightly negatively charged improving their stability in blood circulation, and their size helps to eliminate renal clearance (Luan et al., 2017). They also readily diffuse across plasma membranes (Chen et al., 2016; Matsumoto et al., 2017). Multiple studies have used exosomes to deliver therapeutics to the brain for multiple conditions such as stroke (Li et al., 2020; Venkat et al., 2020), and traumatic brain injury (Chen et al., 2020). Using a zebrafish model, Yang et al. (2015) show that exosomes deliver paclitaxel and doxorubicin (cancer therapeutics) across the BBB. There is a broad potential for exosomes vehicles for the treatment of multiple CNS diseases, with multiple studies previously published but too numerous to discuss, with a few studies are highlighted in Table 2. One of the first studies to demonstrate the use of exosomes to deliver siRNA to the CNS was by Alvarez-Erviti et al. (2011). They demonstrated RVG-targeted exosomes delivered GAPDH siRNA to neurons, microglia, and oligodendrocytes in the brain, inducing in gene knockdown (Alvarez-Erviti et al., 2011). In regards to HIV, Tang et al. (2018) loaded exosomes with HIV Tat protein and these exosomes reactivated primary HIV-infected CD4 + T-cells (Tang et al., 2018). Our laboratory has used microglia-derived exosomes to deliver siRNA across a human BBB model that contains HIV latently infected human telomerase reverse transcriptase immortalized human microglial cells (HTHU-HIV) on the brain side of the model. This study was done to demonstrate a proof of concept that exosomes can transmigrate through the BBB model (Figure 1). We applied microglia-derived exosomes loaded with siRNA specific to Tspan2 to the apical side of the BBB (Reynolds and Mahajan, 2020). Tspan2 siRNA decreases Tspan2 gene expression in HTHU-HIV microglia at the basolateral (brain) side of the BBB model. Knockout of Tspan2 directly led to a decrease in C-X-C motif chemokine 12 (CXCL12) and C-X-C chemokine receptor type 4 (CXCR4) gene expression in HTHU-HIV microglia. The inflammatory response of HTHU latently infected microglia cells was also altered as the gene expression levels of the IL-13 and IL-10 decreases, and gene expression levels for the Fc gamma receptor 2A(FCGR2A) and TNF-α increase (Reynolds and Mahajan, 2020). Overall, this study demonstrates a proof of concept that brain-derived exosomes can be used to cross the BBB to deliver RNA therapeutics to modify genes and proteins that are associated with HIV neuroinflammation. Qiu et al. (2018) elegantly discuss the potential of exosomes to deliver anti-HIV RNA-loaded exosomes (see review Qiu et al., 2018). The potential to use exosomes to delivery HIV therapeutics to the brain is an untapped field and has the potential to deliver therapeutics that reduce inflammatory mediators (inhibitors, siRNA), reduce viral replication (delivery of ARVs), deliver miRNA. However, there are some limitations to using exosomes as drug-delivery vehicles; these limitations are slowly being resolved as the field advances. The heterogenous nature of the content of exosomes, including proteins, lipids, miRNAs, RNA, and DNA, is a primary concern (Record et al., 2011; Ludwig and Giebel, 2012). Loading exosomes from one cell type with a therapeutic and delivering to another cell type is likely to deliver the contents to the latently infected cell type. As described above, miRNA found in exosomes impacts target cells (Hu et al., 2012; Yang et al., 2018). A major limitation of our study described above was in addition to delivering Tspan2 siRNA to microglia cells (Luan et al., 2017; Reynolds and Mahajan, 2020); we likely delivered other contents in exosomes such as DNA, protein, or miRNA. However, we isolated exosomes from microglia cells so we were likely delivering similar contents to the lately infected microglia cells (Reynolds and Mahajan, 2020). One mechanism to overcome this limitation is to use “synthetic exosomes,” such as liposomes or nanoparticles. For example, liposomes conjugated to cell-penetrating peptides increased transfection efficiency of encapsulated plasmid DNA delivery across the BBB (Dos Santos Rodrigues et al., 2019). Our laboratory has shown that Tf-conjugated quantum rods deliver Saquinavir across the BBB to increase CNS concentrations (Mahajan et al., 2010). An excellent review of nanoparticles for delivery of therapeutics for CNS infection nicely describes the potential nanoparticles (please see review DeMarino et al., 2017). An extensive discussion of nanoparticles is beyond the scope of this minireview. Another limitation, in particular with HIV, is the size of viral particles that are similarly sized to the exosome. While isolating exosomes from HIV-infected cells, plasma, serum, or CSF, one may also be concentrating HIV virions. However, Kashanchi’s laboratory (Narayanan et al., 2013; Jaworski et al., 2014; DeMarino et al., 2017; Henderson et al., 2019), a pioneer laboratory in HIV and exosomes, has developed a technique that allows for separation of exosomes from HIV virions. An additional problem with using exosomes as delivery is specific cell targeting. Isolating exosomes from plasma or serum to be used for drug delivery across the BBB does not allow for specific cell type (i.e., astrocytes, microglia) targeting, which is especially important in CNS disease. Several studies have transfected cells to produce exosomes with targeting proteins on their surface or during isolation have enriched for certain proteins (Alvarez-Erviti et al., 2011; El Andaloussi et al., 2013; Sun et al., 2019). Nevertheless, the study of exosomes as drug-delivery vehicles is an exciting field.

**TABLE 2:** Select examples of drug delivery to the CNS.

Source of exosomes	Therapeutic	Citations
**Stroke**		
Bone marrow mesenchymal stem cells	miR-124	Yang et al., 2017
miR-133b^+^ mesenchymal stem cells	miR-133b	Xin et al., 2013
**Alzheimer’s disease**		
Plasma	Quercetin	Qi et al., 2020
Dendritic cell	GADPH siRNA	Alvarez-Erviti et al., 2011
**Parkinson’s disease**		
Macrophage	Catalase	Haney et al., 2015
Blood	Dopamine	Qu et al., 2018
**Glioblastoma**		
U-87	Paclitaxel	Salarpour et al., 2019
Glioblastoma cells	miR-21	Kim et al., 2020

## Discussion

The field of EVs, including exosomes, is a rapidly advancing with significant potential for determining the underpinnings of HIV cellular signaling, transport, and treatment of HAND. Understanding the role that exosomes play in CNS and BBB dysfunction, and neurotoxicity in the CNS that contributes to the progression of HAND, will allow for the development of better treatment modalities, delivery vehicles, and potential biomarkers. Due to their diverse intracellular content, exosomes are poised to provide diagnostic and predictive information that will greatly enhance the development of new therapeutic interventions for neuroinflammation.

## Author Contributions

SM, SK, and JR: concept and writing. NO and HK: writing. All authors contributed to the article and approved the submitted version.

## Conflict of Interest

The authors declare that the research was conducted in the absence of any commercial or financial relationships that could be construed as a potential conflict of interest.
